# Voxel‐based magnetic resonance image postprocessing in epilepsy

**DOI:** 10.1111/epi.13851

**Published:** 2017-07-26

**Authors:** Pascal Martin, Gavin P. Winston, Philippa Bartlett, Jane de Tisi, John S. Duncan, Niels K. Focke

**Affiliations:** ^1^ Department of Neurology and Epileptology Hertie Institute for Clinical Brain Research University of Tübingen Tübingen Germany; ^2^ Department of Clinical and Experimental Epilepsy UCL Institute of Neurology and National Hospital for Neurology and Neurosurgery London United Kingdom; ^3^ Epilepsy Society MRI Unit Chalfont St Peter United Kingdom; ^4^ Clinical Neurophysiology University Clinic Göttingen Germany

**Keywords:** Epilepsy, Voxel‐based, MRI, Postprocessing, MRI negative

## Abstract

**Objective:**

Although the general utility of voxel‐based processing of structural magnetic resonance imaging (MRI) data for detecting occult lesions in focal epilepsy is established, many differences exist among studies, and it is unclear which processing method is preferable. The aim of this study was to compare the ability of commonly used methods to detect epileptogenic lesions in magnetic resonance MRI‐positive and MRI‐negative patients, and to estimate their diagnostic yield.

**Methods:**

We identified 144 presurgical focal epilepsy patients, 15 of whom had a histopathologically proven and MRI‐visible focal cortical dysplasia; 129 patients were MRI negative with a clinical hypothesis of seizure origin, 27 of whom had resections. We applied four types of voxel‐based morphometry (VBM), three based on T1 images (gray matter volume, gray matter concentration, junction map [JM]) and one based on normalized fluid‐attenuated inversion recovery (nFSI). Specificity was derived from analysis of 50 healthy controls.

**Results:**

The four maps had different sensitivity and specificity profiles. All maps showed detection rates for focal cortical dysplasia patients (MRI positive and negative) of >30% at a strict threshold of p < 0.05 (family‐wise error) and >60% with a liberal threshold of p < 0.0001 (uncorrected), except for gray matter volume (14% and 27% detection rate). All maps except nFSI showed poor specificity, with high rates of false‐positive findings in controls. In the MRI‐negative patients, absolute detection rates were lower. A concordant nFSI finding had a significant positive odds ratio of 7.33 for a favorable postsurgical outcome in the MRI‐negative group. Spatial colocalization of JM and nFSI was rare, yet showed good specificity throughout the thresholds.

**Significance:**

All VBM variants had specific diagnostic properties that need to be considered for an adequate interpretation of the results. Overall, structural postprocessing can be a useful tool in presurgical diagnostics, but the low specificity of some maps has to be taken into consideration.

**Key Points**

- All VBM variants show specific diagnostic properties that need to be considered for an adequate interpretation
- VBM based on T_2_‐FLAIR had the best specificity; junction map had the best sensitivity
- VBM based on gray matter volume had the lowest sensitivity, with a low specificity, and appears the least favorable
- Detection of a lesion with normalized FLAIR was associated with a better prognosis for good surgical outcome
- Spatial colocalization of different maps, especially junction map and normalized FLAIR, can improve the confidence in a finding

In the evaluation for epilepsy surgery, magnetic resonance imaging (MRI) has an important role in identifying the structural basis of an epileptogenic network that might be amenable to surgical resection. If the clinical MRI is negative, chances of postoperative seizure freedom are lower than in patients with a visually apparent lesion.^1^, ^2^ Even with optimal methodology, 10–40%^3^, ^4^, ^5^ of presurgically evaluated patients have normal MRI on visual inspection. Although other imaging modalities, such as positron emission tomography (PET), single photon emission computed tomography (SPECT), magnetic, and electrical source imaging can be helpful to infer the localization of an epileptogenic area, and successful surgery is possible in MRI‐negative patients, an intracranial electroencephalography (EEG) is usually necessary, which needs to be targeted and carries expense and risks of morbidity. It is therefore desirable to increase the sensitivity of noninvasive MRI. Focal cortical dysplasia (FCD) is the most common identifiable pathology underlying refractory epilepsy that is not detected on MRI.^6^

Several postprocessing methods have been described, mostly derived from voxel‐based morphometry (VBM).^7^ In contrast to studies in cognitive neuroscience, in which many subjects are compared on a group level, the common approach in clinical epilepsy is to compare a single patient against a group of healthy controls. Although the general utility of voxel‐based postprocessing to improve lesion detection in focal epilepsy is established,^8^ few direct comparative studies exist.^9^ It is unclear which processing method is preferable and how to optimally interpret a VBM analysis, which may be crucial for surgical decision making.

In the present study, we applied recent developments in analytical processing methods such as high‐dimensional spatial normalization (DARTEL)^10^ and improved segmentation/bias correction algorithms, allowing multispectral segmentation in SPM8/SPM12,^11^ and compared four commonly used VBM‐processing routines (classical T_1_‐based VBM methods [gray matter concentration (GMC), gray matter volume (GMV)], the junction map [JM] based on the method of Huppertz et al.,^12^ and the T_2_–fluid‐attenuated inversion recovery [FLAIR]‐based VBM^13^) to compare their ability to detect epileptogenic lesions in 50 healthy controls and 144 patients, comprising a group of MRI‐negative epilepsy patients, some with postoperative results, and a group of MRI‐positive, histopathologically proven FCD patients.

## Methods

### Subjects

We recruited the patients from a consecutive cohort of patients with focal epilepsy who underwent presurgical evaluation at the National Hospital for Neurology and Neurosurgery from 2007 to 2013. Patients were included, if they had (1) a visually evident and histopathologically proven FCD or (2) a normal MRI on visual reporting. A total of 176 patients met these criteria. Patients without a hypothesis on a single lobar level or with divergent results in the presurgical examinations were excluded (32 patients).

Thus, 144 patients (median age = 33 years, range = 17–61, 76 male) were available for the final analysis. Twenty‐seven of 129 MRI‐negative patients had epilepsy surgery, and histopathological data and clinical outcome were available. Fifteen patients had a visually and histopathologically evident FCD. All patients were scanned on the Epilepsy Society 3 Tesla MRI (GE Signa HDx). All scans were acquired with a specific epilepsy protocol and reported by neuroradiologists who specialized in epilepsy. The scanning paradigm consisted of high‐resolution volumetric T_1_‐weighted fast spoiled gradient echo (0.94 × 0.94 × 1.1 mm) and coronal fast spin‐echo T_2_‐FLAIR (0.94 × 0.94 × 5 mm). We also included 50 healthy control subjects (median age = 43 years, range = 19–64) who underwent the same imaging protocol and served as a normal database for all of the processing streams to ensure equal testing conditions. All scans were acquired in the oblique coronal plane perpendicular to the long axis of the hippocampus.

Video‐EEG telemetry was carried out at the National Hospital for Neurology and Neurosurgery and reported by experienced neurophysiologists and neurologists. The results of the voxel‐based image processing were not available at that time and therefore did not influence the evaluation. This study was approved by the research ethics committee of the National Hospital for Neurology and Neurosurgery and the Institute of Neurology as a retrospective evaluation of clinically acquired data.

### MRI processing

All original DICOM images were converted to NIFTI format using MRIConvert (http://lcni.uoregon.edu/~jolinda/MRIConvert/) and processed offline on a Linux‐based workstation. The processing was done using SPM12 (http://www.fil.ion.ucl.ac.uk/spm/) running in MATLAB R2014b (MathWorks, Natick, MA, U.S.A.).

### T_1_‐based stream (VBM and JMs)

This processing stream was based solely on T_1_‐weighted images; T_2_‐FLAIR images were not used. Segmentation was done separately as a single‐channel approach, using the “new segment” algorithm of SPM12 with default settings. We used DARTEL normalization to spatially normalize the gray matter segmentations to Montreal Neurological Institute (MNI) space. We also generated a junction image,^12^ that is, a binary image of the voxels with intensities between the gray matter mean + 0.5 standard deviation (SD) and the white matter mean ± 0.5 SD. We applied the same brain masking as in the normalized FLAIR (nFSI) stream. Finally, the brain masked JMs were smoothed with an 8‐mm full width at half maximum (FWHM) Gaussian kernel.

The gray matter segmentations were separately processed in a conventional VBM approach. The gray matter maps were normalized both with (GMV) and without modulation (GMC). Modulation (i.e., multiplication of the resulting gray matter maps with the Jacobian determinants) was used to account for the effects of spatial normalization and was intended to preserve the absolute GMV. Finally, the normalized gray matter maps were also smoothed with an 8‐mm FWHM Gaussian kernel. For the volume analysis, the total intracranial volume was estimated by summing the gray matter, white matter, and cerebrospinal fluid (CSF) probability maps. This value was used as a covariate of no interest in the general linear model (GLM) analysis to account for global size differences. Because it is difficult to match age in a single subject versus group comparison, we also included age as a covariate of no interest into all GLMs.

### T_2_‐FLAIR–based stream (nFSI)

**Figure 1:**
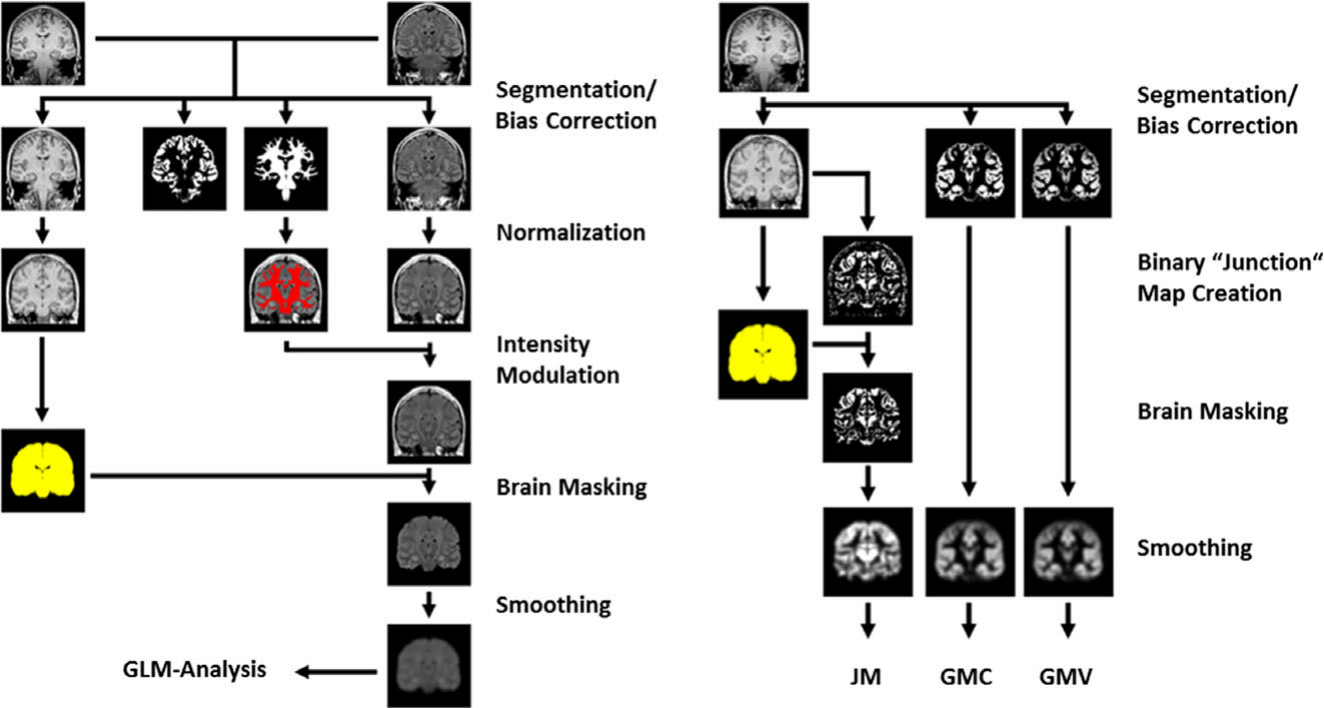
Processing streams for revised T_2_ fluid‐attenuated inversion recovery (FLAIR; left) and T_1_‐FLAIR (right). A schematic processing scheme of T_2_‐FLAIR (normalized; left) and T_1_ images (right) is shown. Images were segmented, spatially and intensity‐normalized, brain masked, smoothed, and finally analyzed using a general linear model (GLM). The right part of the figure shows the processing of T_1_‐weighted images. These were segmented (modulated and unmodulated gray matter segmentation), normalized, and smoothed. Junction maps (JMs) were generated from the bias‐corrected, normalized T_1_‐weighted image, brain masked, and also smoothed. GMC, gray matter concentration; GMV, gray matter volume.

T_2_‐FLAIR images were coregistered to the high‐resolution T_1_‐weighted images using a normalized mutual information cost function in SPM. Both images (T_1_ and coregistered T_2_‐FLAIR) were simultaneously segmented into tissue classes using the multispectral unified segmentation algorithm (new segment) of SPM12 with a default setting (bias regularization = 0.001, bias cutoff = 60 mm).^11^ This procedure also yields a bias‐corrected T_2_‐FLAIR image. This step is a modification of our previous normalized T_2_‐FLAIR signal intensity processing stream^13^ that relied on a separate bias‐correction of T_2_‐FLAIR images. This new approach should improve gray/white matter contrast of T_1_‐weighted images. Visual inspection showed an excellent bias correction and improved segmentation of nonbrain classes (dura, venous sinuses) of this multispectral segmentation compared to a single‐channel approach. The spatial normalization of T_2_‐FLAIR images to MNI space was accomplished with high‐dimensional diffeomorphic normalization (DARTEL normalization).^10^ After spatial normalization, the T_2_‐FLAIR images were intensity‐normalized with the modification that the whole brain white matter (SPM white matter segmentation > 0.5) was used as a reference region.^14^ Figure 1 summarizes the different postprocessing workflows.

The three distinct T_1_ maps and the normalized T_2_‐FLAIR preprocessed images of each subject were then used in a GLM comparing each map against the group of controls at two different significance thresholds:

An uncorrected threshold of p < 0.0001.
A threshold of p < 0.05, corrected for multiple comparisons with the family‐wise error rate (FWE).

We used a two‐tailed t contrast to assess increases and decreases in GMC and volume as signs of focal cortical hypertrophy/atrophy. For JM and normalized T_2_‐FLAIR, we used only a one‐tailed t contrast, as only increase of blurring of the gray/white matter junction and hyperintensity on T_2_‐weighted images are described features of FCDs.^15^

**Figure 2:**
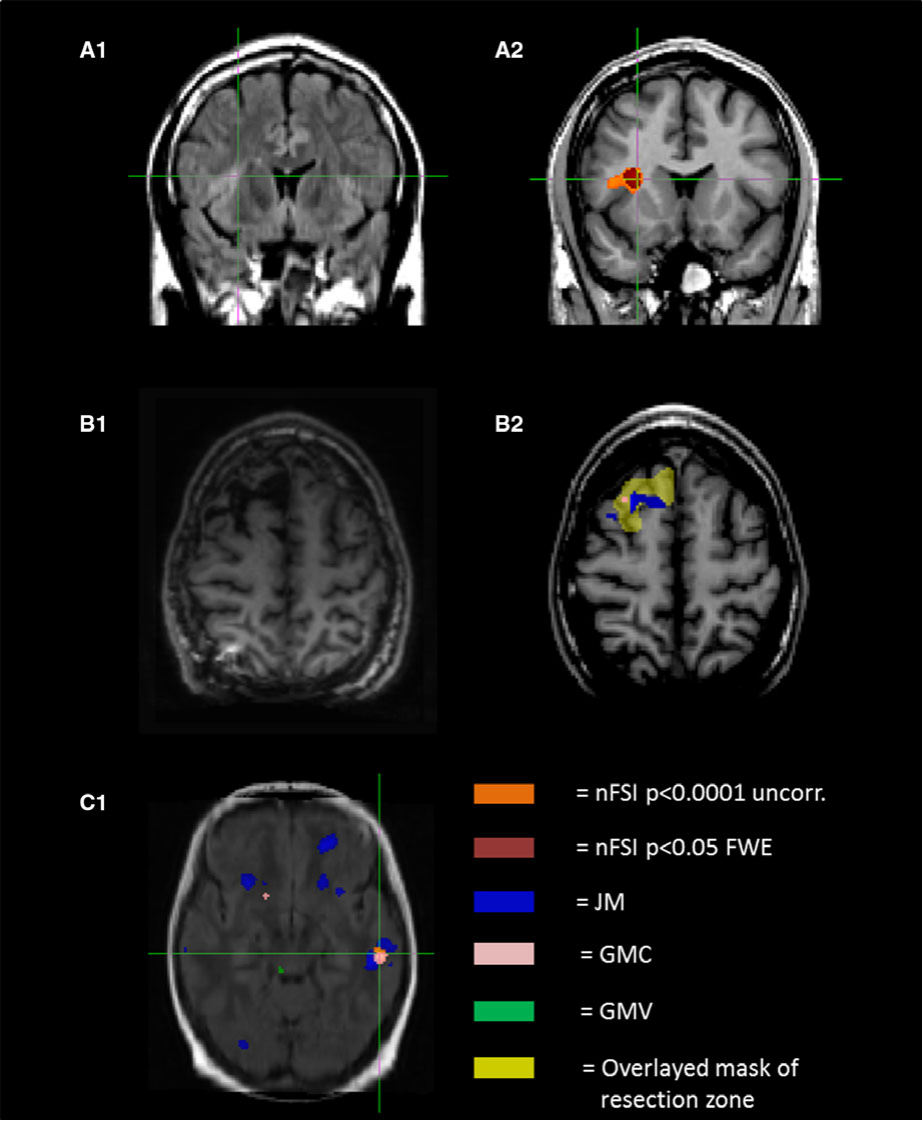
Example of voxel‐based morphometry (VBM) findings. (**A1**) The upper native T_2_–fluid‐attenuated inversion recovery (FLAIR)‐weighted image shows a focal cortical dysplasia (FCD) in the frontal operculum with characteristic hyperintensity and blurring of the gray–white matter zone. (**A2**) The influence of different thresholds on size and amount of VBM findings. Note that the extent of the normalized FLAIR (nFSI) cluster at a threshold of p < 0.0001 (uncorrected [uncorr.]; orange, 471 voxels) is nearly twice as large as the cluster at a threshold of p < 0.05 (family‐wise error rate [FWE]; dark red, 905 voxels) and covers the visible dimension of the lesion more effectively. (**B1**,** B2**) A patient of the magnetic resonance imaging–negative group, who was operated on and histopathology showed FCD type IIa. (**B1**) Postoperative resection. (**B2**) In projection upon this area (yellow), VBM of the preoperative scan shows concordant finding in gray matter concentration (GMC) and junction map (JM). (**C1**) A patient with a hypothesized left temporal epileptogenic focus who did not undergo surgery. A left temporal finding with overlap of three maps (JM, GMC, nFSI), matching the hypothesis, is visualized. **C1** also indicates the high rate of findings discordant with other data (for JM in particular). Note the patchy nonlocalizing distribution of these findings. GMV = gray matter volume.

Figure 2 shows an example visualization of the postprocessing results as well as the influence of different threshold levels.

### Analysis

In a leave‐one‐out strategy (i.e., removing one subject from the control group and testing against the remainder), we visually analyzed each of the 50 control subjects for findings in the four VBM maps (GMC, GMV, JM, nFSI). These were considered to be false positive, and can thus be used to estimate the specificity of the different maps. Specificity was defined as follows:

$$\text{specificity}=\frac{\text{healthy controls without VBM findings (true negative)}}{\text{total number of healthy controls}}.$$

Afterward, we visually analyzed the histopathologically confirmed FCD patients (MRI positive and negative) for suprathreshold clusters in the four different maps in the resected area (true‐positive findings) and outside the resected area. The area resected was determined on postoperative scans, after nonlinear coregistration to the preoperative scans to account for postsurgical brain deformation. For three of the 27 operated patients, no postoperative scans were available, so that we had to assume the whole operated lobe as the resection zone. We subdivided the findings into “concordant with the resection area” (=concordant findings), equivalent to sensitivity, and “discordant with the resection area” (=discordant findings). Sensitivity was derived from all patients with a histopathologically proven FCD (MRI positive and negative). We also calculated the 95% confidence interval (CI) for sensitivity and specificity.^16^ Statistical advice was provided by the Institute for Clinical Epidemiology and Applied Biometry, Tübingen.

For patients without resection, the seizure origin was estimated on a lobar level based on clinical video‐EEG (ictal and interictal EEG, seizure semiology), neuropsychology, and auxiliary examinations (PET, magnetoencephalography [MEG]) as derived from the clinical case conferences. We accepted a bilateral hypothesis, if it could be specified on a lobar level, that is, bitemporal, bifrontal, or bioccipital. One hundred twenty‐six of the 129 patients underwent PET (97.7%), 27 MEG (20.9%), and 15 ictal SPECT (11.6%) to further support the hypothesis of seizure origin. We designated VBM findings in the lobe of hypothesized seizure origin as “concordant with the consensus of other data” and findings outside of the lobe of suspicion as “discordant with the consensus of other data.” Any findings that clearly did not underlie focal epilepsy, for example, intraventricular signal abnormalities due to CSF flow or deep white matter lesions due to small vessel disease, were not considered relevant in any group. Due to the deep location of the insula, it is difficult for scalp EEG to establish the hypothesis of an insular focus and to differentiate insular seizures from frontal, temporal, or parietal lobe seizures.^17^ As such, we, a priori, did not consider the insula as a separate lobe but tried to ascribe the findings to the connected lobes in a clinical context. This scenario became relevant for just a single patient, in whom it was also clinically plausible to declare the insular VBM finding as concordant with a hypothesis in the rolandic area.

We additionally analyzed the spatial overlap of each map with one or more of the other three maps in and outside the suspected lobe; overlapping was defined as at least direct contact of two voxels of different maps.

**Figure 3:**
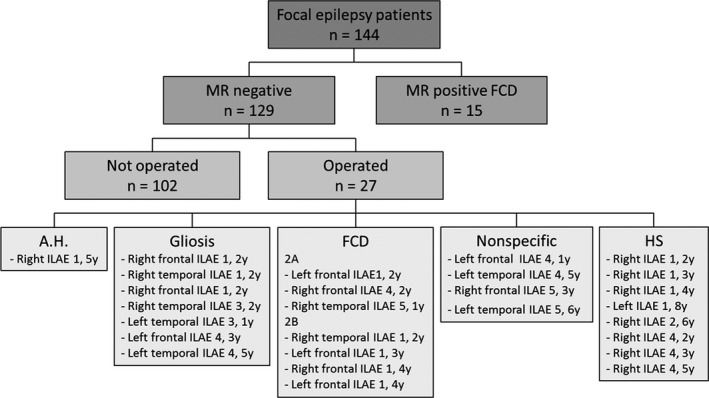
Study tree. This figure gives an overview of the patients included in this study, comprising 144 focal epilepsy patients, including 15 epilepsy patients with a preoperatively diagnosed and postoperatively histopathologically proven focal cortical dysplasia (FCD) and 129 epilepsy patients who had normal magnetic resonance imaging (MRI). Twenty‐seven of these patients had epilepsy surgery, of whom seven had a histopathologically proven FCD, eight had hippocampal sclerosis (HS), seven had gliosis, four were nonspecific and one had amygdala hamartoma (A.H.). The postoperative outcome is indicated based on the International League Against Epilepsy (ILAE) classification 1–6; a good outcome was defined as ILAE classification = 1–2, poor outcome as ILAE classification = 3–6. In total, 14 had a good (ILAE classification = 1–2), 13 a poor (ILAE classification = 3–6) postoperative outcome at 1–7 years (average follow‐up = 3.3 years). The duration of postoperative follow‐up for each patient is indicated following the ILAE classification.

A study tree of the different cohorts is shown in Fig. 3.

## Results

### Healthy controls and specificity

In the healthy controls at a threshold of p < 0.05 (FWE), the false‐positive rates were 10.0–68.0% (Table S1). nFSI had the lowest rate of false‐positive findings with 10.0%, followed by GMV with 34.0%. GMC and JM had higher rates of 58.0% and 68.0%, respectively.

The more liberal threshold of p < 0.0001 (uncorrected) led to a considerably higher number of findings, with up to 100% in GMC and JM. The hierarchy of the four maps remained the same. Colocalization of false‐positive findings in GMC/GMV was most frequent, with each being present in 46.0% of the cases at the low threshold. Other combinations were much less frequent. For p < 0.05 (FWE), only three overlaps were found in total (one each for GMC/JM, GMV/JM, GMC/JM/nFSI). For details, please refer to Table S2.

**Figure 4:**
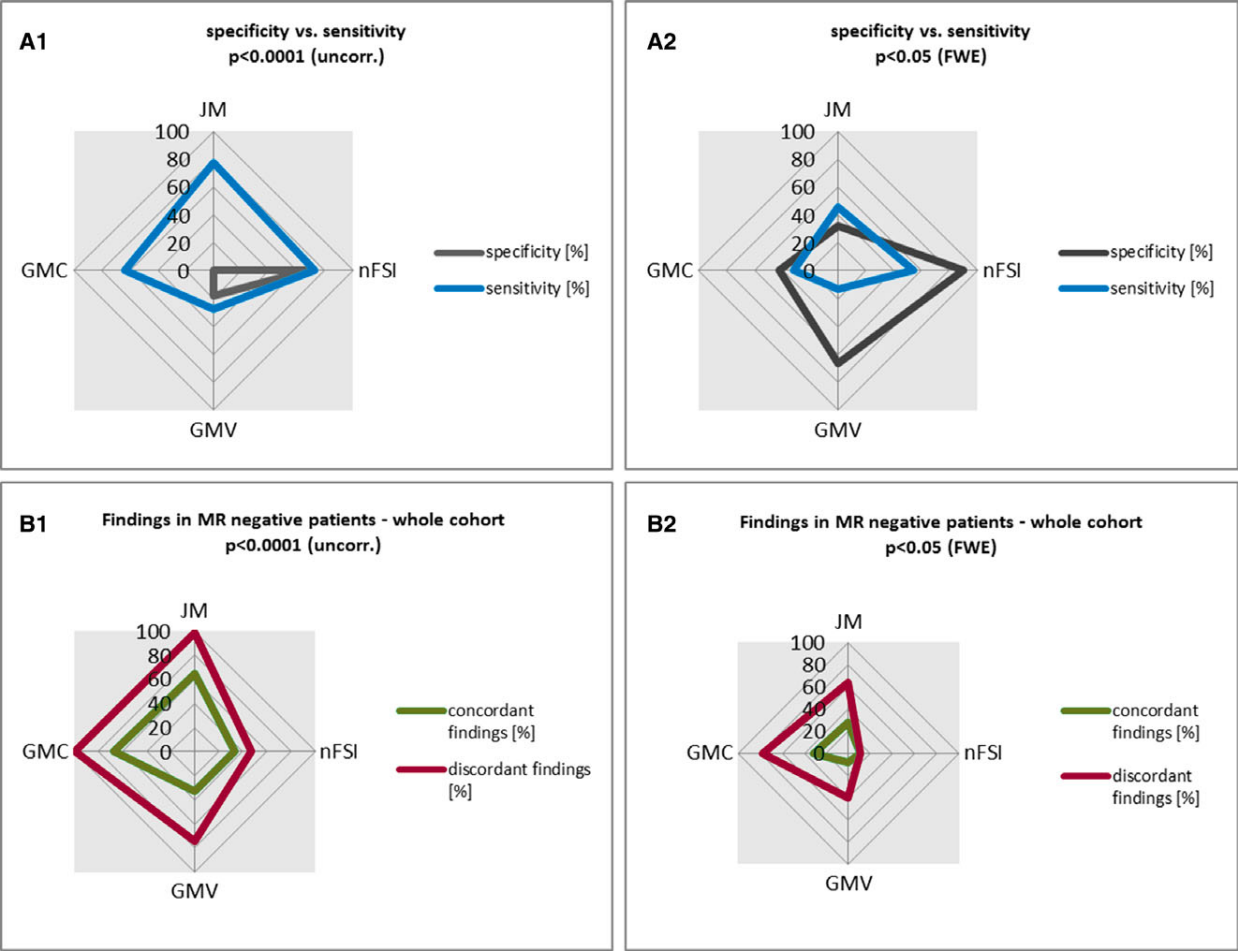
Graphical overview of voxel‐based morphometry (VBM) findings in the different cohorts. This figure summarizes the VBM findings in the different cohorts (healthy controls, magnetic resonance (MRI)‐positive and MRI‐negative focal cortical dysplasia [FCD] patients, MRI‐negative patients) for each analyzed map (gray matter concentration [GMC], gray matter volume [GMV], junction map [JM], nFSI = normalized T_2_–fluid‐attenuated inversion recovery [nFSI]). (**A1**,** B1**) Findings for a threshold of p < 0.0001 (uncorrected [uncorr.]). (**A2, B2**) Findings for a stricter threshold of p < 0.05 (family‐wise error rate [FWE]). Sensitivity (**A1**,** A2**) was derived from true‐positive findings in histopathologically proven FCD patients (n = 22, 15 MRI‐positive, 7 MRI‐negative), specificity (**A1**,** A2**) from findings in healthy controls (n = 50). For MRI‐negative patients (**B1**,** B2**), findings were categorized into those that were concordant with the hypothesis and those that were not (discordant). (**B1**,** B2**) VBM findings in the whole MRI‐negative cohort (n = 129).

Specificity (shown in Fig. 4A1 + 2; see Table S1 for details) for GMC and JM was poor, with 0.0% for p < 0.0001 (uncorrected). nFSI showed the best specificity, with 68.0%. A threshold of p < 0.05 (FWE) led to fewer findings and, consequently, to higher specificity in all maps with the same hierarchy, JM remaining the lowest (32.0%) and nFSI (90.0%) the highest.

### Histopathologically proven FCD group and sensitivity

In the MRI‐positive group of 15 patients with FCD at a threshold of p < 0.05 (FWE), GMC, JM, and nFSI had concordant findings in 40.0%, 46.7%, and 60.0%. GMV, in contrast, had the poorest results, with only 13.3% concordant findings. Discordant findings were absent in nFSI, whereas each of the other maps showed more discordant than concordant findings. A threshold of p < 0.0001 (uncorrected) yielded a higher rate of concordant findings for all four maps. Up to 86.7% of the patients had a concordant finding in JM, and discordant findings increased to up to 100% in JM. For nFSI, the positive ratio of concordant findings remained, but 46.7% discordant findings emerged as well. Please find visualization in Fig. 4A1 + 2 and details in Table S1.

In the seven patients with histopathologically proven FCD but negative MRI, the number of concordant findings was considerably lower for all maps, with a maximum detection rate of 42.9% for JM and nFSI, increasing to 57.1% at p < 0.0001 (uncorrected). NFSI was the only map with more concordant than discordant findings at both thresholds (details are shown in Table S4).

Taking all histopathologically proven FCD patients together, there was sensitivity at p < 0.05 (FWE) of 31.8% for GMC, 45.5% for JM, and 54.5% for nFSI. GMV yielded the poorest results, with 13.6%. For p < 0.0001 (uncorrected), sensitivity increased up to 63.3% in GMC, 77.3% in JM, 72.7% in nFSI, and 27.3% in GMV. Figure 4 illustrates the range of sensitivities.

Concordant colocalizations in multiple maps were rare. The combination of JM/nFSI showed the highest sensitivity of 20.0% and no discordant findings in the MRI‐positive group. In the MRI‐negative group, numbers were smaller, with only one concordant finding per map (equivalent to 14.3% concordant findings). Taken together, the combination of JM/nFSI showed the best specificity, with 18.2% and no discordant findings. Further combinations performed more poorly, especially GMC/GMV, which showed nearly exclusively discordant findings. Details are shown in Table S2.

### MRI‐negative patient group

The detection rates were considerably lower than in the FCD group. The highest values for p < 0.05 (FWE) were found in GMC with 31.8%, followed by JM with 27.9%. nFSI and GMV were 10.1% and 8.5%, respectively. All maps except for nFSI had about three times more discordant than concordant findings.

The more liberal threshold of p < 0.0001 (uncorrected) gave more concordant findings for all four maps, GMC (66.7%), JM (65.1%), nFSI (33.3%), and GMV (32.6%), at the cost of more discordant findings of 99.2% in GMC, 98.4% in JM, 74.4% in GMV, and 47.3% in nFSI. Concordant overlap of maps with more concordant than discordant findings was seen in JM/nFSI, with 6.2% concordant findings in patients. In contrast, discordant findings of GMC/GMV were found in 44.2% of the patients (Table S2).

Separate analysis for temporal and extratemporal hypotheses showed no significant difference at a level of p < 0.05, using two‐tailed Fisher exact test. There was a trend toward more concordant findings between imaging and clinical‐EEG data in extratemporal hypotheses for all maps except for nFSI at p < 0.0001 (uncorrected; see Table S3 for further details).

### Operated subgroup in MRI‐negative patients

**Figure 5:**
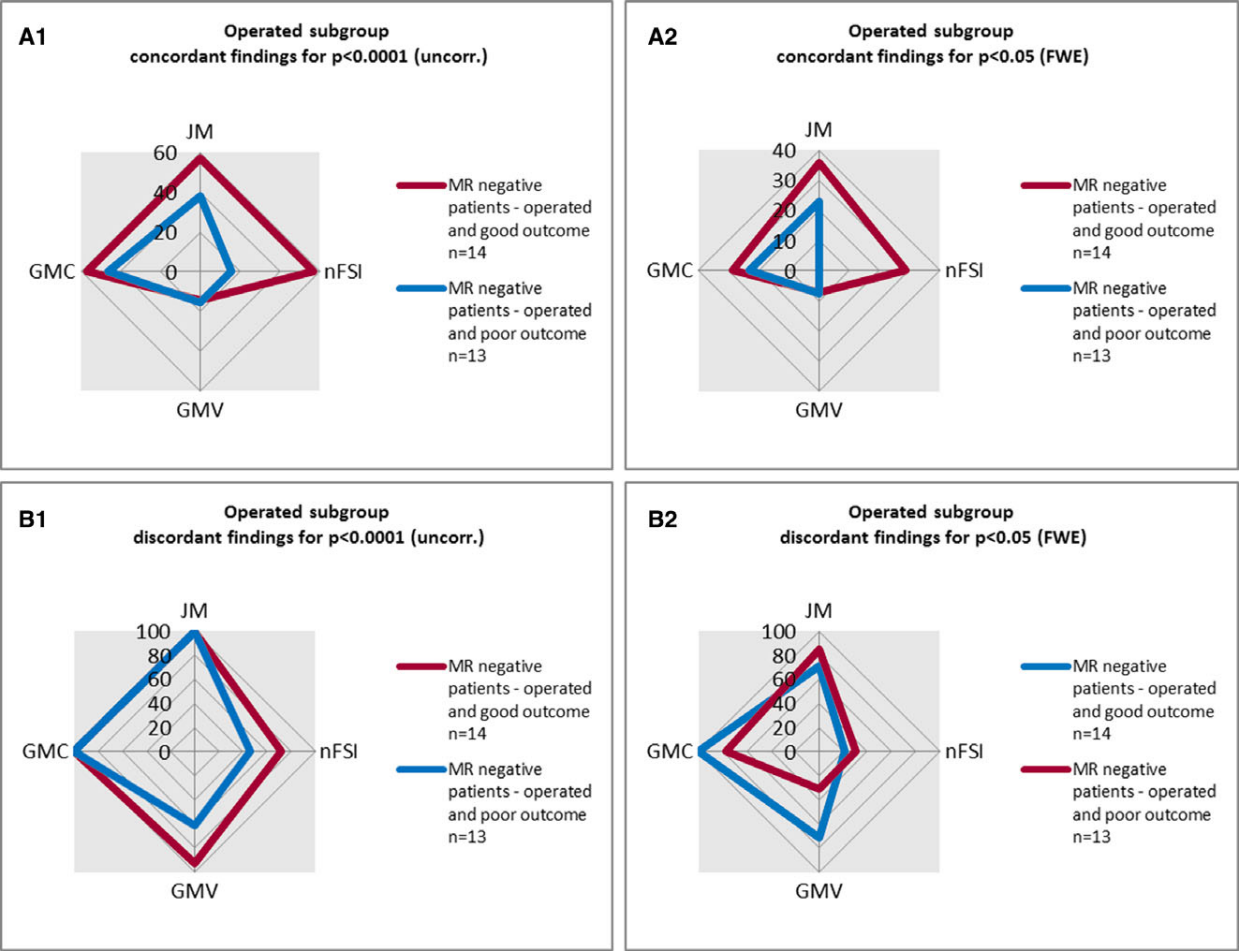
Graphical overview of voxel‐based morphometry (VBM) findings in the subgroup of operated magnetic resonance (MRI)‐negative patients. This figure summarizes the VBM findings in the MRI‐negative subgroups with good (International League Against Epilepsy [ILAE] classification = 1–2) and poor (ILAE classification = 3–5) postsurgical outcome for each map (gray matter concentration [GMC], gray matter volume [GMV], junction map [JM], nFSI = normalized T_2_–fluid‐attenuated inversion recovery [nFSI]) at two different thresholds: (**A1**,** B1**) p < 0.0001 (uncorrected [uncorr.]); (**A2**,** B2**) p < 0.05 (family‐wise error rate [FWE]).

**Figure 6:**
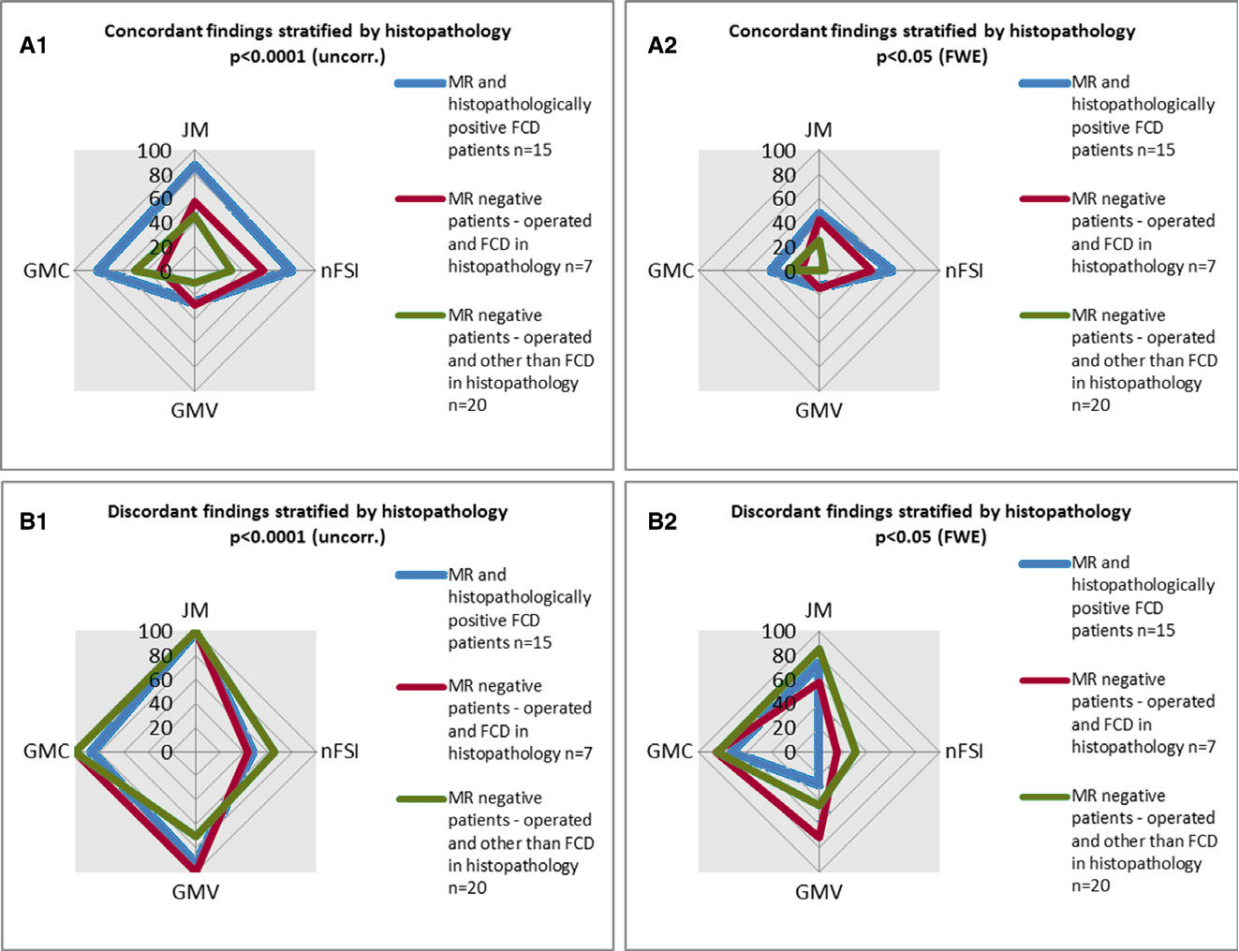
Overview of voxel‐based morphometry (VBM) findings in different histopathological subgroups. This figure summarizes the VBM findings in subgroups of operated patients: patients with focal cortical dysplasia (FCD) and positive magnetic resonance imaging (MRI) (blue), patients with FCD and negative MRI (red), and patients with a histopathological result other than FCD (green; gliosis, hippocampus sclerosis, nonspecific, amygdala hamartia). Each examined map is indexed separately (gray matter concentration [GMC], gray matter volume [GMV], junction map [JM], nFSI = normalized T_2_–fluid‐attenuated inversion recovery [nFSI]). (**A1**,** B1**) Findings for a threshold of p < 0.0001 (uncorrected [uncorr.]). (**A2**,** B2**) Findings for a higher threshold of p < 0.05 (family‐wise error rate [FWE]). Findings were categorized into those that were situated inside the resection area (concordant; **A1**,** A2**) and those lying outside (discordant; **B1**,** B2**).

The analysis of the operated MRI‐negative patients and the further subdivision based on histopathology is shown in Figs. 5 and 6 (detailed numbers in Tables S4 and S5).

Compared to the whole MRI‐negative group of 129 patients, there was a tendency of fewer concordant findings in those patients in whom the histopathological results were nonspecific or gliosis (11 of 27). The FCD subgroup had a higher rate of positive findings (see above).

There was more likely to be a good outcome (International League Against Epilepsy outcome classification groups 1–2^18^), if there was a concordant finding in nFSI at p < 0.0001 (uncorrected; odds ratio = 7.33, 95% CI = 1.16–46.24; Table S6). The odds ratios for other contrasts and thresholds were not significant.

Some colocalizations were also associated with a better outcome, but numbers were low and therefore of limited clinical use (Table S7). JM/nFSI combinations yielded a positive odds ratio of 3.0 at both thresholds.

## Discussion

We compared four variants of VBM, three based on T_1_ (GMV, GMC, JM), and one on normalized T_2_‐FLAIR, for their ability to detect epileptogenic lesions in MRI‐positive, histopathologically proven FCD patients and MRI‐negative epilepsy patients at two different thresholds (p < 0.05 [FWE] and p < 0.0001 [uncorrected]).

### Key findings

The maps had different characteristics concerning specificity in healthy controls and sensitivity in patients with histopathologically proven FCD. All maps showed sensitivity of >30% at a strict threshold of p < 0.05 (FWE) and >60% with a liberal threshold of p < 0.0001 (uncorrected), except for GMV (13.6% and 27.3% detection rate). JM had the highest sensitivity (45–77%), followed by GMC.

Normalized FLAIR had a reasonable balance of sensitivity and specificity for detecting FCD at a threshold of p < 0.05 (FWE; sensitivity of 55%, specificity of 90%). All other maps showed poor specificity, with high rates of false‐positive findings in controls. GMV had the lowest sensitivity, with a low specificity, and so appears to be the least favorable. This poor performance is likely methodologically inherent due to the modulation, which could attenuate focal gray matter differences or introduce global biological variance, for example, due to distributed atrophy.

In the more heterogeneous group of MRI‐negative patients, this profile was similar, but with much fewer concordant findings. Normalized FLAIR only yielded 10–33% concordant findings, and JM 28–65%.

In the subgroup of MRI‐negative, histopathologically proven FCD, VBM performed better than in the whole MRI‐negative group, yet more poorly than in the MRI‐positive FCD group, with 43–57% concordant findings in JM and nFSI, which is as expected given that the MRI‐positive group had visually evident abnormalities.

A good postoperative outcome was significantly positively associated with a concordant finding in JM and especially normalized FLAIR.

Spatial overlap of different maps can improve the confidence in a finding, yet concordant combinations were rare. A combination of JM and normalized FLAIR gave specificity of 100% and sensitivity of 18% for p < 0.05 (FWE).

### Comparison to previous results

Our study compared commonly used VBM methods in preoperative epilepsy diagnostics. Direct comparisons to other studies are limited by technical differences, but we achieved broadly similar results concerning sensitivity and lesion detection. Wang et al.^19^ reported a detection rate of 65% for VBM postprocessing (MAP; includes a JM approach) in a mixed cohort of 150 subtly lesional and nonlesional epilepsy patients, which is comparable to our 65% concordant findings in the MRI‐negative group. They, however, reported a 27% false‐positive rate in 52 healthy controls, which is very different from our finding of 100% at p < 0.0001 (uncorrected) and 68% at p < 0.05 (FWE). As such, we cannot confirm their specificity estimate. This mismatch may be explained by a different approach. They did not use a permutation method, but tested additional healthy controls against a normative database of 150 controls from different scanners, which may result in a bigger variance and, hence, lower statistical significance. Wagner et al.^20^ showed in 91 mostly MRI‐positive patients with histopathologically proven FCD a detection rate of 90% with MAP, which is comparable to our MRI‐positive group with 87% detection for p < 0.0001 (uncorrected). Yet at p < 0.05 (FWE), we found only 46% detections in our mixed group of MRI‐positive and MRI‐negative FCD patients. Smaller studies using MAP gave detection rates of 84–100%^21^, ^22^, ^23^ in MRI‐positive patients, but did not provide information on specificity.

T_1_‐based VBM studies of GMC and/or GMV showed a similar correct detection of lesions at 63–91%,^24^, ^25^, ^26^ but no information on false‐positive findings were available. Salmenpera et al.^9^ came to a similar conclusion concerning GMV, with a low detection rate of seven in 75 (9%).

In a previous cohort, we found similar sensitivity and specificity for normalized FLAIR in MRI‐positive FCD patients (88% vs. 80%)^13^ for p < 0.05 (FWE).

### Statistical thresholds

There is no established threshold in individual postprocessing of MRI data. The applied threshold depends on the clinical question and the characteristics of the particular map. The specific profile of each map needs to be considered when drawing clinical interpretations. In particular, when no threshold or a very liberal threshold is used, there is a risk of overinterpretation. In terms of lesion detection, we compared two cutoffs (p < 0.05 [FWE] and p < 0.0001 [uncorrected]). The threshold of p < 0.05 (FWE) is widely used in various neuroimaging studies and hence can serve as a reference point. However, this correction may be overly strict in the case of subtle lesions. Some studies used more liberal thresholds of p < 0.001 (Pail et al.^24^) or even p < 0.05^27^, ^28^ (Riney et al.,^27^ Braga et al.^28^) without correction. Because in our analysis, GMC and JM already reached a specificity of 0% for p < 0.0001 (uncorrected), we argue that with an even more liberal threshold a positive finding may be very difficult to interpret and hence used p < 0.0001 uncorrected as a liberal threshold.

Also, other technical factors of the analysis can be varied, namely the smoothing kernel/FWHM, the spatial normalization, and voxel resolution. Here, we focused on a common approach using 8‐mm FHWM Gaussian smoothing, 1.5‐mm voxel size, and the state‐of‐the‐art DARTEL normalization. We wanted to assess the profile of fundamentally different VBM approaches/maps and identify their specific profiles. An important aspect is that we did not add visual filtering of the findings, except for those that were clearly spurious, to provide an objective comparison of the different map profiles. Studies that employ visual filtering raise the possibility of observer bias.

### Sensitivity and specificity

It is debatable whether high sensitivity is preferable to high specificity. Sensitivity is important to be sure that no potential lesion has been overlooked, and to act as a screening test to complement visual inspection of MRI data. High specificity helps to increase the confidence in the relevance of a finding. Either way, the specific profile of each map has to be considered. A positive finding in GMC or JM is of little relevance, if it is not in keeping with the electroclinical data. Conversely, the absence of a finding in nFSI will not rule out the presence of a lesion. Although the specificity is low, the presence of a concordant nFSI finding had a positive odds ratio for a good outcome. This is consistent with the better outcome of lesional cases,^26^ including MRI‐negative patients.^19^ These findings should therefore be considered when planning invasive EEG or epilepsy surgery. The handling of discordant findings is, in contrast, more difficult, as some of them could indicate epileptogenic lesions outside the suspected area, for example, in multifocal cases, but also be false positives. Currently, this question cannot be reliably addressed; further studies such as that of Wang et al.,^29^ with longer follow‐up, for example, after reoperations in previously unsuccessful cases, are needed.

### Limitations

Our data analysis was retrospective, and the more robust comparison of VBM findings with surgical outcome and histology of the resection was only possible in 29% of our cohort of 144 patients. The classification for the other MRI‐negative patients depended on the hypothesis that was derived from a best‐available standard in a large tertiary epilepsy center, yet still prone to error and not definitive in many cases. Misclassification of findings are, therefore, likely to occur. Moreover, it is possible that even histopathologically proven FCD patients had multifocal structural abnormalities outside of the resection area, which are not referred to as concordant. Although our patients were recruited from a consecutive cohort, a selection bias was introduced by not including patients with divergent, multifocal, or unclear presurgical evaluations. Therefore, our MRI‐negative results cannot be directly transferred to all MRI‐negative patients, particularly those without any clinical hypothesis.

Given the retrospective nature of our study (with acquisitions from 2007), we had to rely on an anisotropic two‐dimensional (2D) FLAIR dataset that was part of the standard clinical protocol at that time. It is possible that isotropic high‐resolution/3D T_2_‐FLAIR, for example, as used in Adler et al.,^30^ would further improve the results in the FLAIR‐based stream.

Compared to several other studies on lesion detection in epilepsy,^9^, ^24^, ^25^, ^27^ the size of the control group (n = 50) was relatively large. However, we cannot exclude that even larger control group sizes could further improve the results.

### Significance

In summary, our study demonstrates the strengths and limitations of different VBM approaches in epilepsy imaging.

Ongoing work to increase the pickup of subtle structural cerebral abnormalities include postprocessing methods such as surface‐based morphometry, quantitative contrasts (diffusion and perfusion imaging, T_2_‐/T_1_‐relaxometry, magnetization transfer imaging), and use of higher magnetic field strengths. Furthermore, it would be valuable to assess a larger cohort of epilepsy patients from different centers in collaborative efforts. In all VBM approaches, a keen awareness of the importance of specificity as well as sensitivity, and of concordance with clinical and EEG data, is paramount.

## Disclosure

The authors declare no conflicts of interest. We confirm that we have read the Journal's position on issues involved in ethical publication and affirm that this report is consistent with those guidelines.

## Supporting information


**Table S1.** Detailed overview of VBM findings in the different main cohorts.
**Table S2.** Detailed overview of overlapping VBM findings in the different cohorts.
**Table S3.** VBM findings in the MRI‐negative patients, split up into temporal and extratemporal hypothesis.
**Table S4.** Overview of VBM findings in different histopathological subgroups.
**Table S5.** Detailed overview of VBM findings in the subgroup of operated MRI‐negative patients.
**Table S6.** Overview of odds ratios for good versus bad postoperative outcome.
**Table S7.** Overview of odds ratios for good versus bad postoperative outcome for relevant overlap combinations.
