# Supplementary material for: Voxel‐based magnetic resonance image postprocessing in epilepsy
Source: Epilepsia. 2017 Jul 26;58(9):1653–64. doi: 10.1111/epi.13851 (PMC5601223; doi:10.1111/epi.13851)
Supplement: Supplementary file 1 — Table S1. Detailed overview of VBM findings in the different main cohorts. Table S2. Detailed overview of overlapping VBM findings in the different cohorts. Table S3. VBM findings in the MRI‐negative patients, split up into temporal and extratemporal hypothesis. Table S4. Overview of VBM findings in different histopathological subgroups. Table S5. Detailed overview of VBM findings in the subgroup of operated MRI‐negative patients. Table S6. Overview of odds ratios for good versus bad postoperative outcome. Table S7. Overview of odds ratios for good versus bad postoperative outcome for relevant overlap combinations. [file EPI-58-1653-s001.docx]

**Supplements:**

|  |  | Controls n=50 |  |  |  |
| --- | --- | --- | --- | --- | --- |
|  |  | findings [totals/%] |  | specificity [%] | 95% CI [%] |
| p<0.0001(uncorr.) | GMC | 50 / 100.0 |  | 0.0 | 0.0 - 7.1 |
|  | GMV | 41 / 82.0 |  | 18.0 | 8.6-31.4 |
|  | JM | 50 / 100.0 |  | 0.0 | 0.0 - 7.1 |
|  | nFSI | 16 / 32.0 |  | 68.0 | 53.3 - 80.5 |
| p<0.05(FWE) | GMC | 29 / 58.0 |  | 42.0 | 28.2-56.8 |
|  | GMV | 17 / 34.0 |  | 66.6 | 51.2-78.8 |
|  | JM | 34 / 68.0 |  | 32.0 | 19.5 - 46.7 |
|  | nFSI | 5 / 10.0 |  | 90.0 | 78.2 - 96.7 |
|  |  |  |  |  |  |
|  |  | Histopathological positive FCD patients (MR positive and negative) n=22 | | |  |
|  |  | concordant findings [totals/%] | discordant findings [totals/%] | sensitivity [%] | 95% CI [%] |
| p<0.0001(uncorr.) | GMC | 14 / 63.6 | 20 / 90.9 | 63.6 | 40.7-82.8 |
|  | GMV | 6 / 27.3 | 21 / 95.5 | 27.3 | 10.7 - 50.2 |
|  | JM | 17 / 77.3 | 22 / 100.0 | 77.3 | 54.6 - 92.2 |
|  | nFSI | 16 / 72.7 | 10 / 45.5 | 72.7 | 49.8 - 89.3 |
| p<0.05(FWE) | GMC | 7 / 31.8 | 17 / 77.3 | 31.8 | 13.9 - 54.9 |
|  | GMV | 3 / 13.6 | 9 / 40.9 | 13.6 | 2.9-34.9 |
|  | JM | 10 / 45.5 | 15 / 68.2 | 45.5 | 24.4 - 67.8 |
|  | nFSI | 12 / 54.5 | 1 / 4.5 | 54.5 | 32.2 - 75.6 |
|  |  |  |  |  |  |
|  |  | MR negative patients - whole cohort n=129 | |  |  |
|  |  | concordant findings [totals/%] | discordant findings [totals/%] |  |  |
| p<0.0001(uncorr.) | GMC | 86 / 66.7 | 128 / 99.2 |  |  |
|  | GMV | 42 / 32.6 | 96 / 74.4 |  |  |
|  | JM | 84 / 65.1 | 127 / 98.4 |  |  |
|  | nFSI | 43 / 33.3 | 61 / 47.3 |  |  |
| p<0.05(FWE) | GMC | 41 / 31.8 | 100 / 77.5 |  |  |
|  | GMV | 11 / 8.5 | 52 / 40.3 |  |  |
|  | JM | 36 / 27.9 | 82 / 63.6 |  |  |
|  | nFSI | 13 / 10.1 | 14 / 10.9 |  |  |

Supplementary table 1: Detailed overview of VBM findings in the different main cohorts

This table gives detailed information about the VBM findings in the different cohorts (healthy controls; MR positive and negative FCD patients; MR negative patients) for each examined map (GMC=grey matter concentration; GMV=grey matter volume; JM=junction map; nFSI=normalized FLAIR). For patients, findings were categorized into those, which were concordant with the hypothesis or those, who were not (discordant). Specificity was calculated in healthy controls and sensitivity in patients with definite histopathologically proven focal cortical dysplasia (FCD). 95% CI= 95% confidence interval.

|  |  | Controls - n=50 |  |  |  |
| --- | --- | --- | --- | --- | --- |
|  |  | findings [totals/%] |  | specificity [%] | 95% CI [%] |
| p<0.0001(uncorr.) | GMC/GMV | 23 / 46.0 |  | 54.0 | 39.3 - 68.2 |
|  | GMC/JM | 9 / 18.0 |  | 82.0 | 68.6 - 91.4 |
|  | GMC/nFSI | 2 / 4.0 |  | 96.0 | 86.3 - 99.5 |
|  | GMV/JM | 3 / 6.0 |  | 94.0 | 83.5 - 98.8 |
|  | GMV/nFSI | 0 / 0 |  | 100.0 | 92.9 - 100.0 |
|  | JM/nFSI | 1 / 2.0 |  | 98.0 | 89.4 - 100.0 |
|  | GMC/GMV/JM | 3 / 6.0 |  | 94.0 | 83.5 - 98.8 |
|  | GMC/GMV/nFSI | 1 / 2.0 |  | 98.0 | 89.4 - 100.0 |
|  | GMC/JM/nFSI | 1 / 2.0 |  | 98.0 | 89.4 - 100.0 |
|  | GMV/JM/nFSI | 0 / 0 |  | 100.0 | 92.9 - 100.0 |
|  | GMC/GMV/JM/nFSI | 2 / 4.0 |  | 96.0 | 86.3 - 99.5 |
| p<0.05(FWE) | GMC/GMV | 0 / 0 |  | 100.0 | 92.9 - 100.0 |
|  | GMC/JM | 1 / 2 |  | 98.0 | 89.4 - 100.0 |
|  | GMC/nFSI | 0 / 0 |  | 100.0 | 92.9 - 100.0 |
|  | GMV/JM | 1 / 2 |  | 98.0 | 89.4 - 100.0 |
|  | GMV/nFSI | 0 / 0 |  | 100.0 | 92.9 - 100.0 |
|  | JM/nFSI | 0 / 0 |  | 100.0 | 92.9 - 100.0 |
|  | GMC/GMV/JM | 0 / 0 |  | 100.0 | 92.9 - 100.0 |
|  | GMC/GMV/nFSI | 0 / 0 |  | 100.0 | 92.9 - 100.0 |
|  | GMC/JM/nFSI | 1 / 2 |  | 98.0 | 89.4 - 100.0 |
|  | GMV/JM/nFSI | 0 / 0 |  | 100.0 | 92.9 - 100.0 |
|  | GMC/GMV/JM/nFSI | 0 / 0 |  | 100.0 | 92.9 - 100.0 |
|  |  |  |  |  |  |
|  |  | Histopathological positive FCD patients (MR positive and negative) n=22 | | | |
|  |  | concordant findings [totals/%] | discordant findings [totals/%] | sensitivity [%] | 95% CI [%] |
| p<0.0001(uncorr.) | GMC/GMV | 2 / 9.1 | 11 / 50.0 | 9.1 | 1.1 - 29.2 |
|  | GMC/JM | 2 / 9.1 | 3 / 13.6 | 9.1 | 1.1 - 29.2 |
|  | GMC/nFSI | 1 / 4.5 | 3 / 13.6 | 4.5 | 0.1 - 22.8 |
|  | GMV/JM | 1 / 4.5 | 0 / 0 | 4.5 | 0.1 - 22.8 |
|  | GMV/nFSI | 0 / 0 | 1 / 13.6 | 0.0 | 0.0 - 15.4 |
|  | JM/nFSI | 4 / 18.2 | 0 / 0 | 18.2 | 5.2 - 40.3 |
|  | GMC/GMV/JM | 0 / 0 | 1 / 13.6 | 0.0 | 0.0 - 15.4 |
|  | GMC/GMV/nFSI | 0 / 0 | 0 / 0 | 0.0 | 0.0 - 15.4 |
|  | GMC/JM/nFSI | 2 / 9.1 | 1 / 13.6 | 9.1 | 1.1 - 29.2 |
|  | GMV/JM/nFSI | 1 / 4.5 | 0 / 0 | 4.5 | 0.1 - 22.8 |
|  | GMC/GMV/JM/nFSI | 2 / 9.1 | 0 / 0 | 9.1 | 1.1 - 29.2 |
| p<0.05(FWE) | GMC/GMV | 0 / 0 | 4 / 18.2 | 0.0 | 0.0 - 15.4 |
|  | GMC/JM | 0 / 0 | 0 / 0 | 0.0 | 0.0 - 15.4 |
|  | GMC/nFSI | 0 / 0 | 0 / 0 | 0.0 | 0.0 - 15.4 |
|  | GMV/JM | 1 / 4.5 | 0 / 0 | 4.5 | 0.1 - 22.8 |
|  | GMV/nFSI | 0 / 0 | 0 / 0 | 0.0 | 0.0 - 15.4 |
|  | JM/nFSI | 4 / 18.2 | 0 / 0 | 18.2 | 5.2 - 40.3 |
|  | GMC/GMV/JM | 0 / 0 | 0 / 0 | 0.0 | 0.0 - 15.4 |
|  | GMC/GMV/nFSI | 0 / 0 | 0 / 0 | 0.0 | 0.0 - 15.4 |
|  | GMC/JM/nFSI | 2 / 9.1 | 0 / 0 | 9.1 | 1.1 - 29.2 |
|  | GMV/JM/nFSI | 0 / 0 | 0 / 0 | 0.0 | 0.0 - 15.4 |
|  | GMC/GMV/JM/nFSI | 1 / 4.5 | 0 / 0 | 4.5 | 0.1 - 22.8 |
|  |  |  |  |  |  |
|  |  | MR negative patients - whole cohort n=129 | | | |
|  |  | concordant findings [totals/%] | discordant findings [totals/%] |  |  |
| p<0.0001(uncorr.) | GMC/GMV | 14 / 10.9 | 57 / 44.2 |  |  |
|  | GMC/JM | 8 / 6.2 | 22 / 17.1 |  |  |
|  | GMC/nFSI | 5 / 3.9 | 12 / 9.3 |  |  |
|  | GMV/JM | 1 / 0.8 | 4 / 3.1 |  |  |
|  | GMV/nFSI | 2 / 1.6 | 6 / 4.7 |  |  |
|  | JM/nFSI | 8 / 6.2 | 5 / 3.9 |  |  |
|  | GMC/GMV/JM | 0 / 0 | 14 / 10.9 |  |  |
|  | GMC/GMV/nFSI | 1 / 0.8 | 7 / 5.4 |  |  |
|  | GMC/JM/nFSI | 4 / 3.1 | 4 / 3.1 |  |  |
|  | GMV/JM/nFSI | 0 / 0 | 0 / 0 |  |  |
|  | GMC/GMV/JM/nFSI | 1 / 0.8 | 3 / 2.3 |  |  |
| p<0.05(FWE) | GMC/GMV | 3 / 2.3 | 15 / 11.6 |  |  |
|  | GMC/JM | 1 / 0.8 | 3 / 2.3 |  |  |
|  | GMC/nFSI | 1 / 0.8 | 4 / 3.1 |  |  |
|  | GMV/JM | 1 / 0.8 | 0 / 0 |  |  |
|  | GMV/nFSI | 0 / 0 | 0 / 0 |  |  |
|  | JM/nFSI | 1 / 0.8 | 1 / 0.8 |  |  |
|  | GMC/GMV/JM | 0 / 0 | 3 / 2.3 |  |  |
|  | GMC/GMV/nFSI | 0 / 0 | 0 / 0 |  |  |
|  | GMC/JM/nFSI | 4 / 3.1 | 1 / 0.8 |  |  |
|  | GMV/JM/nFSI | 0 / 0 | 0 / 0 |  |  |
|  | GMC/GMV/JM/nFSI | 0 / 0 | 2 / 1.6 |  |  |

Supplementary table 2: Detailed overview of overlapping VBM findings in the different cohorts

This table gives detailed information about the overlapping VBM findings in the different cohorts (healthy controls; MR positive and negative FCD patients; MR negative patients) in every combination of the four used maps (GMC=grey matter concentration; GMV=grey matter volume; JM=junction map; nFSI=normalized FLAIR). For patients, the findings were categorized by those who fit the hypothesis (concordant) or those who did not (discordant). Based on this, specificity was calculated in healthy controls and sensitivity in patients with definite histopathologically proven focal cortical dysplasia (FCD).

|  |  | MR negative patients - temporal hypothesis n=84 | |
| --- | --- | --- | --- |
|  |  | concordant findings [totals/%] | discordant findings  [totals/%] |
| p<0.0001(uncorr.) | GMC | 52 / 61.9 | 83 / 98.8 |
|  | GMV | 23 / 27.4 | 61 / 72.6 |
|  | JM | 51 / 60.7 | 82 / 97.6 |
|  | nFSI | 28 / 33.3 | 41 / 48.8 |
| p<0.05(FWE) | GMC | 25 / 29.8 | 65 / 77.4 |
|  | GMV | 6 / 7.1 | 30 / 35.7 |
|  | JM | 21 / 25.0 | 51 / 60.7 |
|  | nFSI | 06.07.2001 | 10.7 |
|  |  |  |  |
|  |  | MR negative patients - extratemporal hypothesis n=45 | |
|  |  | concordant findings [totals/%] | discordant findings  [totals/%] |
| p<0.0001(uncorr.) | GMC | 34 / 75.6 | 45 / 100.0 |
|  | GMV | 19 / 42.2 | 35 / 77.8 |
|  | JM | 33 / 73.3 | 45 / 100.0 |
|  | nFSI | 15 / 33.3 | 20 / 44.4 |
| p<0.05(FWE) | GMC | 16 / 35.6 | 35 / 77.8 |
|  | GMV | 5 / 11.1 | 22 / 48.9 |
|  | JM | 15 / 33.3 | 31 / 68.9 |
|  | nFSI | 7 / 15.6 | 3 / 21.4 |
|  |  |  |  |
|  |  | MR negative patients - temporal vs. extratemporal | |
|  |  |  | Fisher's exact test |
| p<0.0001(uncorr.) | GMC | concordant | p=0.17 |
|  |  | discordant | p=1 |
|  | GMV | concordant | p=0.11 |
|  |  | discordant | p=0.67 |
|  | JM | concordant | p=0.18 |
|  |  | discordant | p=0.54 |
|  | nFSI | concordant | p=1 |
|  |  | discordant | p=0.71 |
| p<0.05(FWE) | GMC | concordant | p=0.55 |
|  |  | discordant | p=1 |
|  | GMV | concordant | p=0.51 |
|  |  | discordant | p=0.19 |
|  | JM | concordant | p=0.41 |
|  |  | discordant | p=0.44 |
|  | nFSI | concordant | p=0.22 |
|  |  | discordant | p=1 |

Supplementary table 3: VBM findings in the MR negative patients, split up into temporal and extratemporal hypothesis

This table summarizes the VBM findings in the MRI negative patients analyzed separately for temporal and extratemporal hypothesis. Each examined map is indexed separately (GMC=grey matter concentration; GMV=grey matter volume; JM=junction map; nFSI=normalized FLAIR). Findings were categorized into those, which were situated inside the hypothesized area (concordant) and those lying outside (discordant). Fishers exact test indicates the difference between the two groups.

|  |  | MR negative patients - operated and different than FCD in histopathology n=20 | | | |
| --- | --- | --- | --- | --- | --- |
|  |  | concordant findings [totals/%] | discordant findings [totals/%] |  |  |
| p<0.0001(uncorr.) | GMC | 10 / 50.0 | 20 / 100.0 |  |  |
|  | GMV | 2 / 10.0 | 14 / 70.0 |  |  |
|  | JM | 9 / 45.0 | 20 / 100.0 |  |  |
|  | nFSI | 6 / 30.0 | 13 / 65.0 |  |  |
| p<0.05(FWE) | GMC | 5 / 25.0 | 17 / 85.0 |  |  |
|  | GMV | 0 / 0 | 9 / 45.0 |  |  |
|  | JM | 5 / 25.0 | 17 / 85.0 |  |  |
|  | nFSI | 1 / 5.0 | 6 / 30.0 |  |  |
|  |  |  |  |  |  |
|  |  | MR and histopathological positive FCD patients n=15 | |  |  |
|  |  | concordant findings [totals/%] | discordant findings [totals/%] |  |  |
| p<0.0001(uncorr.) | GMC | 12 / 80.0 | 13 / 86.7 |  |  |
|  | GMV | 4 / 26.7 | 14 / 93.3 |  |  |
|  | JM | 13 / 86.7 | 15 / 100.0 |  |  |
|  | nFSI | 12 / 80.0 | 7 / 46.7 |  |  |
| p<0.05(FWE) | GMC | 6 / 40.0 | 11 / 73.3 |  |  |
|  | GMV | 2 / 13.3 | 4/ 26.7 |  |  |
|  | JM | 7 / 46.7 | 11 / 73.3 |  |  |
|  | nFSI | 9 / 60.0 | 0 / 0 |  |  |
|  |  |  |  |  |  |
|  |  | MR negative patients - operated and FCD in histopathology n=7 | | |  |
|  |  | concordant findings [totals/%] | discordant findings [totals/%] |  |  |
| p<0.0001(uncorr.) | GMC | 2 / 28.6 | 7 / 100.0 |  |  |
|  | GMV | 2 / 28.6 | 7 / 100.0 |  |  |
|  | JM | 4 / 57.1 | 7 / 100.0 |  |  |
|  | nFSI | 4 / 57.1 | 3 / 42.9 |  |  |
| p<0.05(FWE) | GMC | 1 / 14.3 | 6 / 85.7 |  |  |
|  | GMV | 1 / 14.3 | 5 / 71.4 |  |  |
|  | JM | 3 / 42.9 | 5 / 57.1 |  |  |
|  | nFSI | 3 / 42.9 | 1 / 14.3 |  |  |

Supplementary table 4: Overview of VBM findings in different histopathological subgroups

This table summarizes the VBM findings in subgroups of operated patients: Patients with a histopathological result different than FCD (gliosis, hippocampus sclerosis, non-specific, amygdala hamartia), patients with FCD and positive MRI, patients with FCD and negative MRI. Each examined map is indexed seperatly (GMC=grey matter concentration; GMV=grey matter volume; JM=junction map; nFSI=normalized FLAIR). Findings were categorized into those, which were situated inside the resection area (concordant) and those lying outside (discordant).

|  |  | MR negative patients - operated n=27 | |
| --- | --- | --- | --- |
|  |  | concordant findings [totals/%] | discordant findings [totals/%] |
| p<0.0001(uncorr.) | GMC | 14 / 51.9 | 27 / 100.0 |
|  | GMV | 4 / 14.8 | 21 / 77.8 |
|  | JM | 13 / 48.2 | 27 / 100.0 |
|  | nFSI | 10 / 37.0 | 16 / 59.3 |
| p<0.05(FWE) | GMC | 7 / 25.9 | 24 / 88.9 |
|  | GMV | 2 / 7.4 | 14 / 51.9 |
|  | JM | 8 / 29.6 | 21 / 77.8 |
|  | nFSI | 4 / 14.8 | 7 / 25.9 |
|  |  |  |  |
|  |  | MR negative patients - operated and good outcome n=14 | |
|  |  | concordant findings [totals/%] | discordant findings [totals/%] |
| p<0.0001(uncorr.) | GMC | 8 / 57.1 | 14 / 100.0 |
|  | GMV | 2 / 14.3 | 13 / 92.9 |
|  | JM | 8 / 57.1 | 14 / 100.0 |
|  | nFSI | 8 / 57.1 | 10 / 71.4 |
| p<0.05(FWE) | GMC | 4 / 28.6 | 14 / 100.0 |
|  | GMV | 1 / 7.1 | 10 / 71.4 |
|  | JM | 5 / 35.7 | 10 / 71.4 |
|  | nFSI | 4 / 28.6 | 3 / 21.4 |
|  |  |  |  |
|  |  | MR negative patients - operated and poor outcome n=13 | |
|  |  | concordant findings [totals%] | discordant findings [totals/%] |
| p<0.0001(uncorr.) | GMC | 6 / 46.2 | 13 / 100.0 |
|  | GMV | 2 / 15.4 | 8 / 61.5 |
|  | JM | 5 / 38.5 | 13 / 100.0 |
|  | nFSI | 2 / 15.4 | 6 / 46.2 |
| p<0.05(FWE) | GMC | 3 / 23.1 | 10 / 76.9 |
|  | GMV | 1 / 7.7 | 4 / 30.8 |
|  | JM | 3 / 23.1 | 11 / 84.6 |
|  | nFSI | 0 / 0 | 4 / 30.8 |

Supplementary table 5: Detailed overview of VBM findings in the subgroup of operated MR negative patients

This table summarizes the VBM findings in the subgroup of operated, MR negative patients for each analyzed map (GMC=grey matter concentration; GMV=grey matter volume; JM=junction map; nFSI=normalized FLAIR). Findings were categorized into those, which were located inside the resection area (concordant) and those lying outside (discordant).

|  |  | MR negative patients - operated n=27 | |
| --- | --- | --- | --- |
|  |  | Odds ratio | 95% CI |
| p<0.0001(uncorr.) | GMC | 1.56 | 0.34 - 7.11 |
|  | GMV | 0.92 | 0.11 - 7.67 |
|  | JM | 2.13 | 0.46 - 9.94 |
|  | nFSI | 7.33 | 1.16 - 46.24 |
| p<0.05(FWE) | GMC | 1.33 | 0.24 - 7.56 |
|  | GMV | 0.92 | 0.05 - 16.46 |
|  | JM | 1.86 | 0.34 - 10.05 |
|  | nFSI | 11.57 | 0.56 - 239.75 |

Supplementary table 6: Overview of odds ratio good vs. bad postoperative outcome

This table shows the odds ratio for good postoperative outcome (defined as ILAE 1-2) indicated by a concordant VBM finding in the operated region for each map (GMC=grey matter concentration; GMV=grey matter volume; JM=junction map; nFSI=normalized FLAIR). The 95% confidence interval (CI) is also shown.

|  |  | MR negative patients - operated n=27 | |
| --- | --- | --- | --- |
|  |  | Odds ratio | 95% CI |
| p<0.0001(uncorr.) | GMC/GMV | 0.29 | 0.01 - 7.70 |
|  | GMC/JM | 0.93 | 0.02 - 50.30 |
|  | GMC/nFSI | 0.29 | 0.01 - 7.70 |
|  | GMV/JM | 0.29 | 0.01 - 7.70 |
|  | GMV/nFSI | 0.93 | 0.02 - 50.30 |
|  | JM/nFSI | 3.00 | 0.11 - 80.40 |
|  | GMC/GMV/JM | 0.93 | 0.02 - 50.30 |
|  | GMC/GMV/nFSI | 0.93 | 0.02 - 50.30 |
|  | GMC/JM/nFSI | 0.93 | 0.02 - 50.30 |
|  | GMV/JM/nFSI | 0.93 | 0.02 - 50.30 |
|  | GMC/GMV/JM/nFSI | 3.00 | 0.11 - 80.40 |
| p<0.05(FWE) | GMC/GMV | 0.93 | 0.02 - 50.30 |
|  | GMC/JM | 0.93 | 0.02 - 50.30 |
|  | GMC/nFSI | 0.93 | 0.02 - 50.30 |
|  | GMV/JM | 0.29 | 0.01 - 7.70 |
|  | GMV/nFSI | 0.93 | 0.02 - 50.30 |
|  | JM/nFSI | 3.00 | 0.11 - 80.40 |
|  | GMC/GMV/JM | 0.93 | 0.02 - 50.30 |
|  | GMC/GMV/nFSI | 0.93 | 0.02 - 50.30 |
|  | GMC/JM/nFSI | 3.00 | 0.11 - 80.40 |
|  | GMV/JM/nFSI | 0.93 | 0.02 - 50.30 |
|  | GMC/GMV/JM/nFSI | 0.93 | 0.02 - 50.30 |

Supplementary table 7: Overview of odds ratio good vs. bad postoperative outcome for relevant overlap combinations

This table shows the odds ratio for good postoperative outcome (defined as ILAE 1-2) indicated by a concordant co-localizing finding of different maps in the operated region (GMC=grey matter concentration; GMV=grey matter volume; JM=junction map; nFSI=normalized FLAIR). The 95% confidence interval (CI) is also shown.
